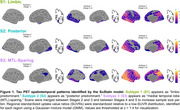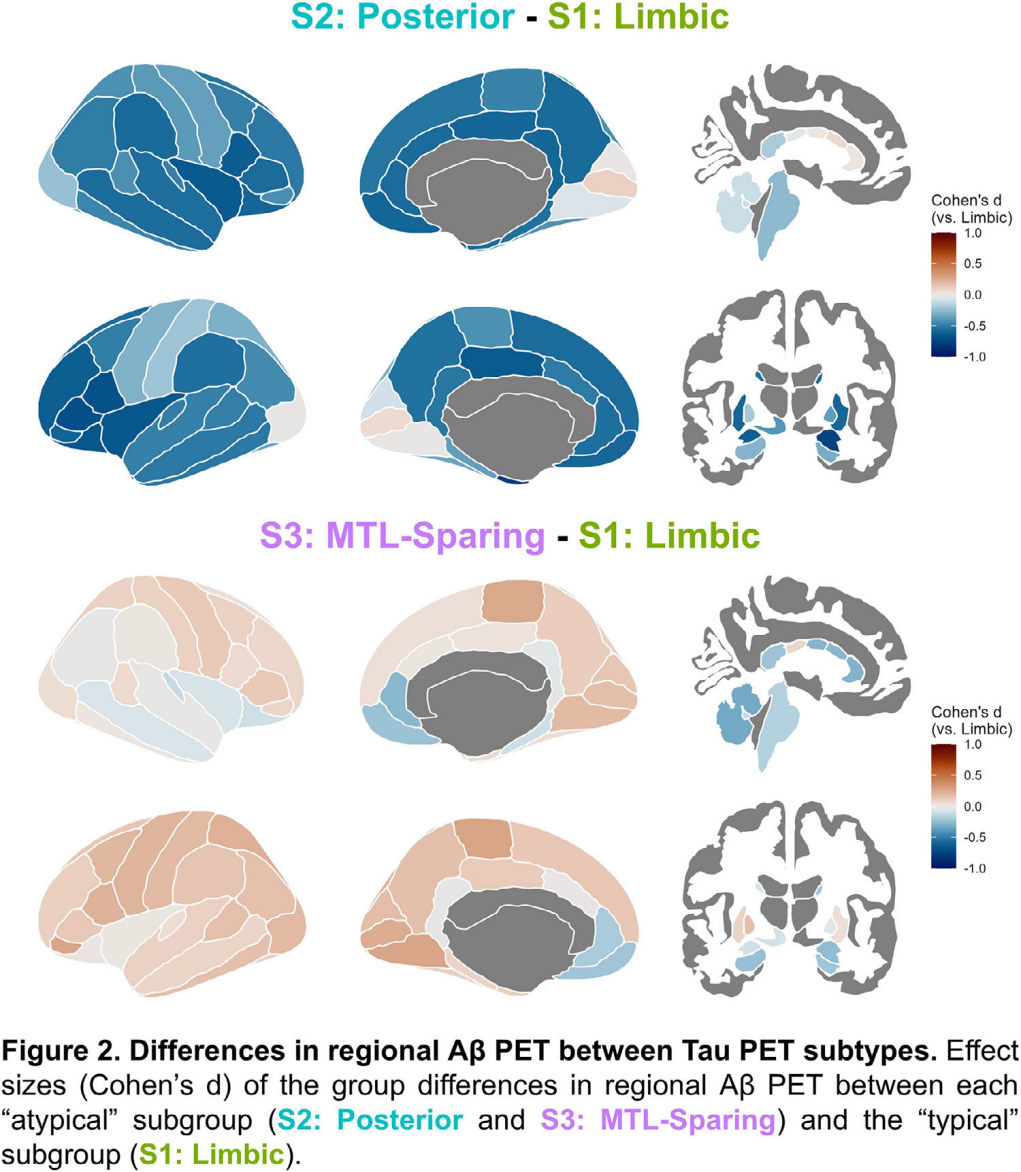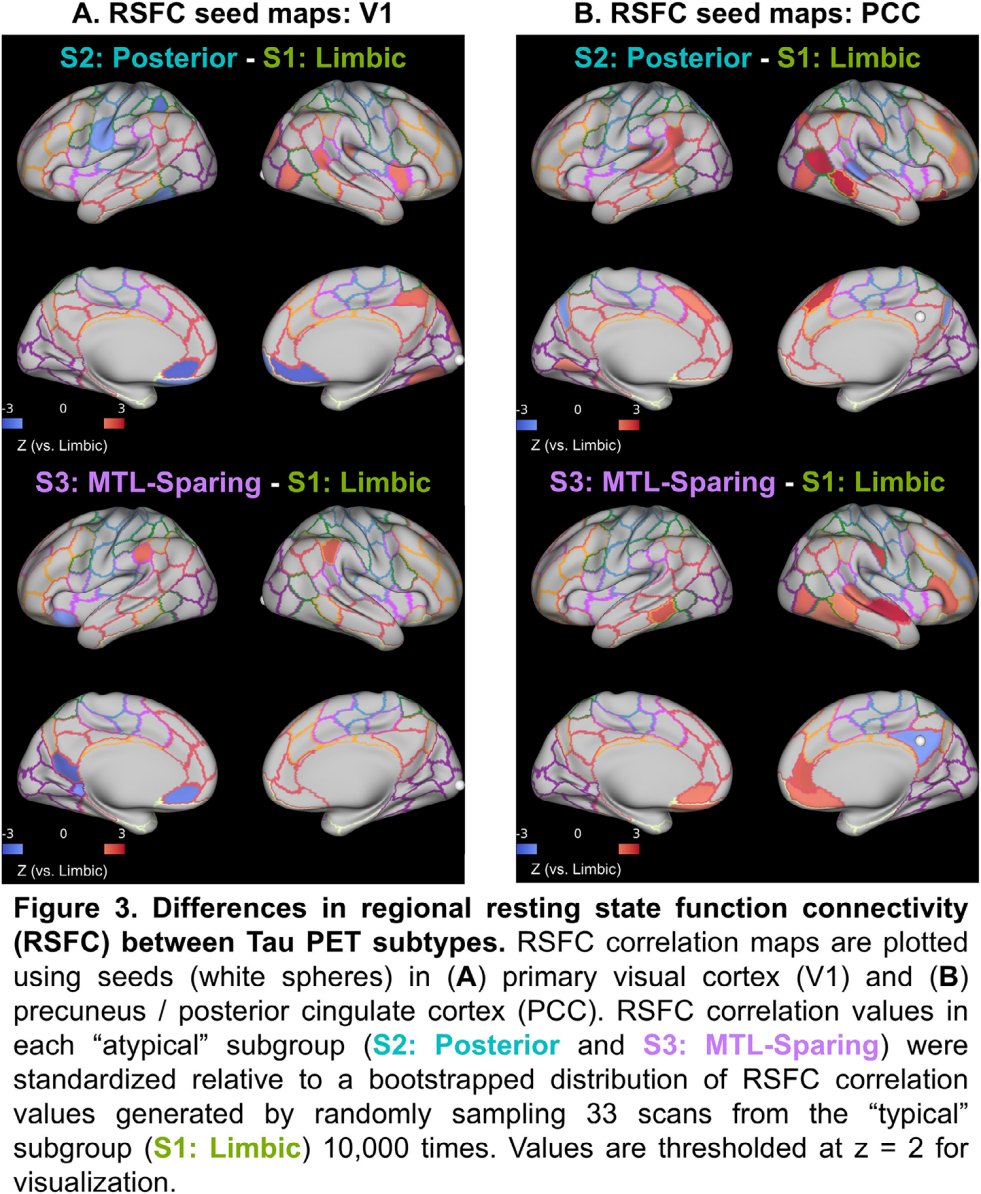# Differing network patterns of functional connectivity and amyloid PET between tau PET subtypes

**DOI:** 10.1002/alz70856_097884

**Published:** 2025-12-24

**Authors:** Peter R Millar, Stephanie Doering, Babatunde Adeyemo, Nicole S. McKay, Nicholas V Metcalf, June Roman, Pete Canfield, Saurabh Jindal, Shaney Flores, Jeremy F. Strain, Julie K. Wisch, Jason J. Hassenstab, Suzanne E. Schindler, John C. Morris, Brian A. Gordon, Beau Ances, Tammie L.S. Benzinger

**Affiliations:** ^1^ Department of Neurology, Washington University School of Medicine, St. Louis, MO, USA; ^2^ Washington University in St. Louis, St. Louis, MO, USA; ^3^ Washington University in St. Louis School of Medicine, St. Louis, MO, USA; ^4^ Washington University in St. Louis, School of Medicine, St. Louis, MO, USA; ^5^ Washington University School of Medicine, St. Louis, MO, USA; ^6^ Washington University School of Medicine in St. Louis, St. Louis, MO, USA; ^7^ Washington University School of Medicine, Saint Louis, MO, USA

## Abstract

**Background:**

Spatiotemporal patterns of tau accumulation differ between distinct Alzheimer disease (AD) phenotypes and can be identified with PET imaging. Tau accumulation may be driven in part by trans‐neuronal spreading through network connections, which can be estimated using resting state functional connectivity MRI (RSFC), and by amyloid‐β (Aβ) deposition. We aimed to replicate tau PET subtypes and tested subtype‐specific differences in network patterns of RSFC and Aβ PET.

**Method:**

We used the Subtype and Stage Inference (SuStaIn) model to identify spatiotemporal patterns in 820 tau PET scans (^18^F‐flortaucipir) from 629 Knight ADRC participants (mean age = 70.3, SD = 8.6, N = 690 CDR® 0, N = 130 CDR > 0). RSFC correlations were analyzed using seeds in primary visual cortex (V1) and precuneus/posterior cingulate cortex (PCC). Aβ PET SUVRs using ^11^C‐Pittsburgh‐compound‐B (PiB, *N* = 365) or ^18^F‐florbetapir (FBP, *N* = 423) were harmonized to Centiloid.

**Result:**

We identified three tau PET subtypes, resembling previously reported patterns: limbic predominant (*N* = 157), posterior predominant (*N* = 27), and medial temporal lobe (MTL)‐sparing (*N* = 52), Figure 1. The remaining scans were tau‐negative (*N* = 570) or had unstable classifications (*N* = 14). MTL‐sparing participants were younger, more severely impaired, and had greater levels of Aβ PET than other groups (*p*'s < 0.001). Posterior predominant participants were less frequently *APOE* ε4 carriers than limbic predominant participants (*p*'s < 0.001). Although posterior predominant participants exhibited lower levels of Aβ PET than limbic predominant participants in anterior regions and global summaries, Aβ PET was slightly greater in occipital regions, Figure 2. Compared to limbic predominant participants, posterior predominant participants exhibited stronger RSFC correlations between V1 and adjacent regions within the visual network and other networks, Figure 3. MTL‐sparing participants exhibited stronger RSFC correlations between PCC and other default mode regions throughout the cortex.

**Conclusion:**

We replicated three distinct spatiotemporal subtypes of tau PET accumulation and their associations with individual differences and AD severity in an independent sporadic AD cohort. We identified novel differences in spatial patterns of Aβ PET and RSFC, which may contribute to or result from unique patterns of tau spreading within these subtypes.